# Pioneering Advances in Veterinary Medicine: From Diagnosis to Treatment

**DOI:** 10.3390/ani15040516

**Published:** 2025-02-12

**Authors:** Felisbina Luisa Queiroga

**Affiliations:** 1 Department of Veterinary Sciences, University de Trás-os-Montes e Alto Douro (UTAD), 5000-801 Vila Real, Portugal; fqueirog@utad.pt; 2 Animal and Veterinary Research Centre (CECAV), University de Trás-os-Montes e Alto Douro (UTAD), 5000-801 Vila Real, Portugal

Continuous advancements in veterinary medicine have significantly improved the health and longevity of companion animals while also contributing to wildlife conservation efforts. Over the last few decades, innovations in diagnostic techniques and treatment approaches have revolutionized veterinary practice. These developments not only enhance disease management in animals but also provide valuable insights into zoonotic diseases, reinforcing the One Health perspective. This Special Issue presents a collection of studies that explore various aspects of the diagnosis and treatment of animal diseases, offering new scientific evidence in both companion and wild animal medicine. The included articles highlight the refinement of diagnostic techniques, the identification of novel biomarkers, and the development of innovative therapeutic approaches.

The first study by Magliocca et al. [1] explores the molecular detection of viral and bacterial pathogens in red foxes (*Vulpes vulpes*) from Italy, highlighting the role of wildlife in disease transmission. The authors identified several viral and bacterial agents, including *Protoparvovirus carnivoran 1* (PPVC-1), *Canine adenovirus* (CAdV-1 and CAdV-2), *Canine circovirus* (Canine CV), and *Leptospira* spp. The findings suggest a potential risk of cross-species transmission and highlight the need for continued surveillance in wildlife populations. Similarly, a study on non-invasive wildlife disease surveillance using real-time PCR in the endangered Iberian desman demonstrates how molecular tools enhance disease monitoring while supporting conservation [2]. Focusing on the endangered Iberian desman (*Galemys pyrenaicus*), Ripa et al. [2] utilized non-invasive sampling and real-time PCR assays to detect parasitic and bacterial infections. The study provided baseline data for conservation efforts, demonstrating the effectiveness of molecular techniques in wildlife disease monitoring.

Advancements in diagnostic techniques in companion animals are also emphasized. A study on hemogram-derived inflammatory markers in cats with chronic kidney disease by Krofič Žel et al. [3] explored the potential of hemogram-derived inflammatory markers, such as the neutrophil-to-lymphocyte ratio (NLR) and platelet-to-lymphocyte ratio (PLR), as indicators of systemic inflammation in feline chronic kidney disease (CKD). The results showed a correlation between these markers and disease severity, suggesting their potential as non-invasive tools for monitoring disease progression. The study by Medardo et al. [4] evaluated the diagnostic performance of cytology versus bacterial culture in identifying septic exudative effusions in veterinary patients. While bacterial culture remains the gold standard, cytology was found to provide rapid preliminary results that could guide early clinical decision-making. The study by Licenziato et al. [5] focused on alternative therapeutic targets for canine B-cell lymphoma, specifically investigating the use of the drugs BI2536 and MZ1 to indirectly modulate Myc expression. The results demonstrated promising anti-cancer effects in vitro, paving the way for further studies on targeted therapies. Lastly, a review on next-generation sequencing (NGS) techniques in veterinary medicine by Domrazek & Jurka [6], discussed the growing role of NGS in veterinary diagnosis, covering applications such as pathogen identification, cancer genomics, and genetic predisposition studies, illustrating how genomic tools are shaping personalized treatments.

Innovative treatment approaches are also explored, including a study evaluating medical-grade honey and *Hypericum perforatum* for second-intention wound healing in cats. Chatzimisios et al. [7] demonstrated that although both treatments improved tissue perfusion, the honey-treated wounds exhibited superior histological parameters, suggesting potential clinical applications for wound management. Similarly, a review on mesenchymal stem cells (MSCs) by Picazo et al. [8], provided an overview of MSCs’ applications for treating wounds, ocular diseases and neuromuscular disorders in companion animals. This study discussed the therapeutic mechanisms of MSCs and outlined challenges in standardizing protocols for their clinical use. Additionally, a study by Mitsui & Uchida [9] on gallbladder erosion/ulcers and hemocholecyst in dogs provided new clinical and pathological insights into a poorly documented condition. The results suggest a link between gallbladder erosion and COX-1/COX-2 expression, contributing to our understanding of biliary disorders in animals.

Finally, imaging advancements are discussed in a review by Ercolin et al. [10] on novel ultrasonographic techniques for detecting neoplasms in dogs and cats, emphasizing the role of contrast-enhanced ultrasonography and elastography as non-invasive alternatives for oncology diagnostics. These techniques were found to improve the differentiation between benign and malignant tumors, offering non-invasive alternatives for cancer detection.

In conclusion, this Special Issue provides high-quality scientific contributions that enhance our understanding of disease epidemiology, diagnostic methodologies, and innovative treatments in veterinary medicine. We hope that these studies will inspire further research and clinical applications, ultimately improving the health and welfare of both companion and wild animals while strengthening the interconnectedness of human, animal, and environmental health.
